# Ukgansan Protects Dopaminergic Neurons against MPTP-Induced Neurotoxicity via the Nurr1 Signaling Pathway

**DOI:** 10.1155/2022/7393557

**Published:** 2022-09-20

**Authors:** In-Cheol Chae, Jung-Hee Jang, In-Chan Seol, Yoon-Sik Kim, Gunhyuk Park, Ho-Ryong Yoo

**Affiliations:** ^1^Department of Cardiology and Neurology, College of Korean Medicine, Daejeon University, Daejeon 34520, Republic of Korea; ^2^KM Science Research Division, Korea Institute of Oriental Medicine, 1672 Yuseongdae-ro, Yuseong-gu, Daejeon 34054, Republic of Korea; ^3^Herbal Medicine Resources Research Center, Korea Institute of Oriental Medicine, 111 Geonjae-ro, Naju-si, Jeollanam-do 58245, Republic of Korea

## Abstract

Nuclear receptor-related 1 protein (Nurr1) is a nuclear hormone receptor that protects dopaminergic neurons and is a promising therapeutic target for Parkinson's disease (PD). Parkinson's disease is a neurodegenerative disorder caused by the destruction of dopaminergic neurons in the substantia nigra pars compacta (SNpc), and the long-term use of conventional dopamine replacement therapies causes many side effects, highlighting the need for new treatments such as complementary and alternative medicine. Ukgansan has been used in East Asia to treat neurological disorders, including neurodegenerative diseases, and has been reported to have strong effects in treating patients with PD. In addition, recent studies have reported that Ukgansan has a neuroprotective potential. However, there are no detailed studies on the mechanism of action of Nurr1. Thus, unlike previous studies, we focused on the Nurr1 pathways. We confirmed neurotoxicity and apoptosis signaling in the differentiated PC12 cells. In addition, to confirm the protective effect of Ukgansan, we conducted behavioral tests (motor coordination and postural balance, and bradykinesia) and tyrosine hydroxylase immunohistochemistry in both the SNpc and striatum. Specifically, this study demonstrated the effect of Ukgansan in protecting dopaminergic neurons and increasing Nurr1 involved in maintaining dopamine levels by activating Nurr1 expression in MPTP-induced PC12 cells and a mouse model of PD. In this mechanism, the loss of dopaminergic neurons and dopamine depletion were suppressed, and motor impairment caused by 1-methyl-4-phenyl-1,2,3,6-tetrahydropyridine toxicity was improved. These results provide evidence that Ukgansan ameliorates PD's motor symptoms and progression.

## 1. Introduction

Parkinson's disease (PD) is a progressive disease caused by degeneration or loss of dopaminergic neurons in the substantia nigra pars compacta (SNpc), presenting symptoms including rigidity and tremors at rest, bradykinesia, and postural instability [1, 2]. Various factors induce neurodegeneration in PD, such as inflammation, oxidative stress, mitochondrial dysfunction, and cellular apoptosis; however, PD etiology is still not fully elucidated. Although various treatments are being used and new attempts are being made to treat PD, the gold standard for treating PD is dopamine supplementation with L-DOPA [2, 3]. Dopamine replacement therapies can temporarily improve PD symptoms but cause side effects in patients with PD, such as motor, autonomic, genitourinary, and gastrointestinal disorders [3]. Therefore, new therapies that can slow or stop PD progression are constantly needed. As a result, patients with PD are resorting to complementary and alternative medicine (CAM), with those who have experienced CAM at least once ranging from 25.7%–76% worldwide [4]. This indicates that due to the limitations of conventional therapy, many patients with PD are interested in using CAM to treat its motor and non-motor symptoms [4, 5].

Nuclear receptor-related 1 protein (Nurr1), also called NR4A2, is a transcription factor belonging to the nuclear receptor family [6]. Nurr1 is essential for the differentiation and maintenance of dopaminergic neuronal function in the midbrain and also plays an important role in regulating the expression of tyrosine hydroxylase (TH), vesicular monoamine transporter 2 (VMAT2), and dopamine transporters (DAT) in dopaminergic neurons [6, 7]. Based on the mechanism by which Nurr1 increases the expression of dopamine-related genes, protects dopaminergic neurons from neurotoxins, inhibits microglial activity, and reduces neuroinflammation, Nurr1-based therapies that activate or upregulate Nurr1 may be beneficial in PD treatment [6, 8]. A clinical study reported that Nurr1 mRNA levels in peripheral blood mononuclear cells (PBMCs) increased when PD patients took dopamine agonists, such as pramipexole [9].

Ukgansan (Yi-Gan san in China or Yokukansan in Japan), is a traditional herbal medicine that first appeared in “Bao Ying Cuo Yao,” and has been observed to have therapeutic effects on neurodegenerative diseases such as PD and behavioral and psychological symptoms of dementia (BPSD) in various dementias such as Alzheimer's disease (AD), dementia with Lewy bodies (LBD) and frontotemporal dementia (FTD) [10–14]. According to a retrospective study using data for 5 years conducted at a Korean medicine hospital in South Korea, herbal medicine modified with Ukgansan is the most frequently used to improve the motor symptoms of PD patients [15]. Furthermore, in clinical studies with PD patients, it was reported that Ukgansan improved BPSD or neuropsychiatric symptoms, including hallucinations, anxiety, and apathy without side effects, and was a useful herbal medicine for drug-induced parkinsonism [16, 17]. In addition, in laboratory experiments, Ukgansan was reported to have neuroprotective effects through catechol-O-methyltransferase (COMT)-inhibition and antioxidant response, and improved movement impairment induced by neurotoxicity in dopaminergic neurons [18–21].

However, no studies have revealed the effect and mechanism of Ukgansan on the therapeutic effect of PD by regulating the Nurr1 signaling pathway. Therefore, this study was conducted to investigate the protective effects of Ukgansan against 1-methyl-4-phenyl-1,2,3,6-tetrahydropyridine (MPTP)-induced neurotoxicity in mice and to explore the underlying mechanisms of Nurr1 action.

## 2. Materials and Methods

### 2.1. Chemicals

Paraformaldehyde (PFA), 3,3-diaminobenzidine (DAB), sodium chloride, sucrose, ethanol, Histomount medium, dimethyl sulfoxide (DMSO), nerve growth factor (NGF), MPTP, 1-methyl-4-phenylpyridinium (MPP+), hydrogen peroxide, phosphate-buffered saline (PBS), and sodium citrate buffer were purchased from Sigma-Aldrich (St. Louis, MO, USA). Roswell Park Memorial Institute (RPMI) medium, penicillin/streptomycin, and fetal bovine serum (FBS) were purchased from Gibco (MD, USA). Biotinylated goat anti-rabbit antibody, goat anti-mouse antibody, normal goat serum (NGS), and the VECTASTAIN Elite ABC Kit were purchased from Vector Laboratories (Burlingame, CA, USA). Rabbit anti-TH antibody was purchased from Millipore (Bedford, MA, USA). Rabbit anti-Nurr1 antibody was purchased from OriGene (Rockville, MD, USA). The Nurr1 ELISA Kit was purchased from Biomatik (Wilmington, DE, USA). The 2-CAT (N-D) Research ELISA Kit was purchased from Rocky Mountain Diagnostics (Colorado Springs, CO, USA). All other reagents were of analytical grade.

### 2.2. Preparation of Ukgansan Extracts

Ukgansan was obtained from the Department of Korean Medicine at Daejeon University for the researcher's use. Ukgansan comprises seven herbs: Atractylodis Rhizoma, Poria Sclerotium, Cnidii Rhizoma, Angelicae Gigantis Radix, Uncariae Ramulus cum Uncus, Bupleuri Radix, and Glycyrrhizae Radix et Rhizoma, at a ratio of 4 : 4 : 3 : 3 : 3 : 2 : 1.5, respectively (Table 1). The herbs were extracted using boiling water for 2 h. The extract was filtered, evaporated on a rotary vacuum evaporator, and lyophilized using a freeze-dryer (yield, 29.8%). The resulting powder was stored at 4°C until further use.

### 2.3. Differentiated PC12 Cells' Culture

The PC12 cell line, pheochromocytoma, derived from the rat adrenal medulla, was obtained from the Korean Cell Line Bank (#21721). PC12 cells were maintained in an RPMI medium supplemented with 10% heat-inactivated FBS and 1% penicillin/streptomycin in an atmosphere of 95% air and 5% CO_2_ at 37°C. The culture medium was changed every 2–3 days. Cultured PC12 cells were treated with NGF for 7 days, with fresh medium and reagents supplied every 24 h. All experiments were conducted 12 h after the cells were seeded on 24-well plates at a density of 2 × 10^4^ (PC12 cells).

### 2.4. Measurement of Cytotoxicity

Cell viability was evaluated using a Cell Counting Kit (CCK-8; Dojindo, Kumamoto, Japan) according to the manufacturer's protocol. Briefly, cells were seeded in 96-well plates at a density of 1 or 2 × 10^4^ cells/well and treated with Ukgansan (0–200 *μ*g/mL) for 1 h, followed by stimulation with MPP+ (500 *μ*M) for an additional 23 h. The CCK-8 reagent was added to each well, and the mixture was incubated for 4 h. Absorbance was read at 450 nm using the SpectraMax i3 Multi-Mode Detection Platform (Molecular Devices, Sunnyvale, CA, USA).

### 2.5. Animals

Male C57BL/6 mice (8 weeks old, 23-24 g, purchased from RaonBio, Yongin, South Korea) were maintained under temperature- and light-controlled conditions (20–23°C, 12-h light/12-h dark cycle) and allowed ad libitum access to food and water. All animals were acclimatized for seven days prior to drug administration. The experimental protocol was approved by the Institutional Animal Care Committee of Daejeon University (DJUARB2021-018) and was performed according to the Animal Care and Use Committee of Daejeon University guidelines.

### 2.6. Drug Administration

The mice were assigned to one of four groups: (1) control, (2) MPTP, (3) MPTP + Ukgansan 200 mg/kg/day, and (4) MPTP + ropinirole 1 mg/kg/day. Ukgansan, dissolved in normal saline, was administered for five days consecutively. The dose of ropinirole was selected based on a previous study of the effect of ropinirole on MPTP-treated C57BL/6 mice. The control group was administered an equal volume of normal saline during the same period. On day 3 of Ukgansan treatment, MPTP (20 mg/kg; dissolved in saline) was injected intraperitoneally four times at 2 h intervals. Equal volumes of vehicles (0.25 mL) were assigned to the control group.

### 2.7. Pole Test

We performed pole tests 3 and 7 days after the last MPTP injection. The mice were held on top of the pole (diameter, 8 mm; height, 55 cm; six rough surfaces). The time required for the mice to turn down completely was recorded as the time to turn (T-turn). The time that mice needed to descend with all four feet on the floor was recorded as the time for locomotion activity (T-LA), with a 60 s cut-off limit for each trial.

### 2.8. Brain Tissue Preparation

Seven days after MPTP treatment, mice were anesthetized immediately and perfused transcardially with 0.05 M PBS, followed by cold 4% PFA in 0.1 M phosphate buffer. Brains were removed and post-fixed in 0.1 M phosphate buffer containing 4% PFA at 4°C overnight and immersed in a solution containing 30% sucrose in 0.05 M PBS for cryoprotection. Serial 30 *μ*m-thick coronal sections were cut on a freezing microtome (Leica Instruments GmbH, Nussloch, Germany) and stored in cryoprotectant (25% ethylene glycol, 25% glycerol, and 0.05 M phosphate buffer) at 4°C until used for the immunohistochemistry (IHC) study. For western blotting and kit-based analyses, mice were decapitated, and their brains were isolated and stored at −80°C until use.

### 2.9. Immunohistochemistry (IHC) Analysis

For IHC analysis, the brain sections were briefly rinsed in PBS and treated with 1% hydrogen peroxide for 15 min. Sections were incubated with rabbit anti-TH (1 : 1000) overnight at 4°C in the presence of 0.3% Triton X-100 and NGS. After rinsing in PBS, sections were incubated with biotinylated anti-rabbit IgG (1 : 200) for 90 min, rinsed, and incubated with ABC (1 : 100) for 1 h at room temperature. The peroxidase activity was visualized by incubating sections with DAB in 0.05 M Tris-buffered saline (TBS, pH 7.6). After rinsing with PBS several times, sections were mounted on gelatin-coated slides, dehydrated, and coverslipped using Histomount medium. Images were photographed at 40× and 100× magnifications using an optical light microscope (Olympus Microscope System BX53; Olympus, Tokyo, Japan) equipped with a 20× objective lens.

### 2.10. Western Blotting

For western blotting and ELISA kit-based analyses, the cell or SNpc were rapidly homogenized and centrifuged using standard laboratory techniques. The final supernatants were stored at −80°C until use. For the detection of Nurr1 proteins, the supernatants were lysed with protein extraction buffer for whole protein analysis. The lysates were separated by 15% SDS-PAGE and were then transferred to a membrane. The membranes were incubated with 5% skim milk in Tris-buffered-saline (TBST) for 1 h. Then they were incubated with primary antibody overnight at 4°C, after which they were incubated with HRP-conjugated secondary antibody IgG for 1 h. Immunoreactive bands were detected using an ECL detection kit and visualized with a LAS-3000 mini system (Fujifilm Corporation, Tokyo, Japan).

Then, Nurr1 levels of the SNpc of the mouse brain were assessed using a commercially available fluorometric assay kit, following the protocol described by the manufacturer (Biomatik).

### 2.11. Dopamine, TH, VMAT2, DAT, and Cleaved Caspase-3 Levels

Dopamine content in the striatum (ST) of the mouse brain was assessed using a commercially available fluorometric assay kit, following the protocol described by the manufacturer (BioVision; Milpitas, CA, USA). TH, VMAT2, and DAT levels were determined using ELISA kits, following the manufacturer's instructions (MyBioSource; San Diego, CA, USA). In addition, cleaved caspase-3 levels were determined using ELISA kits following the manufacturer's instructions (Cell Signaling; Beverly, MA, USA).

### 2.12. Statistical Analyses

All statistical parameters were calculated using GraphPad Prism 5.0 software (GraphPad Software, San Diego, CA, USA). Values are expressed as mean ± standard error of the mean (S.E.M.). Statistical comparisons between different treatments were performed using one-way analysis of variance (ANOVA) with Tukey's multiple comparison test. Statistical significance was set at *P* < 0.05.

## 3. Results

### 3.1. Inhibitory Effects of Ukgansan on MPP+-Induced Neurotoxicity and Programmed Apoptosis Activity in Differentiated PC12 Cells

**Figure 1:**
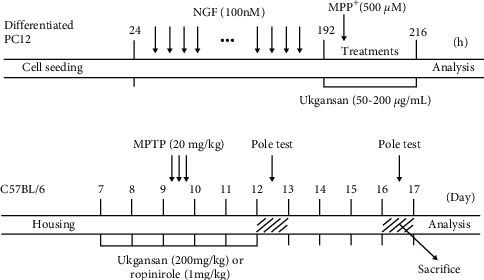
Protective effects of Ukgansan against MPP+-induced neurotoxicity in differentiated PC12 cells. Differentiated PC12 cells were treated with Ukgansan for 1 h and MPP+ for 23 h and then the cell viability was measured using the CCK-8 assay. (a) The effect of Ukgansan on MPP+-induced programmed apoptosis signaling. Its factors (cleaved caspase-3) were measured by ELISA kits. (b) Values are given as the mean ± S.E.M. ^*∗∗∗*^*p* < 0.001 compared to the control group, and ^###^*p* < 0.001 compared to the MPP+-toxicity group.

To assess the protective effects of Ukgansan on differentiated PC12 cells, we performed a CCK-8 assay. Treatment with MPP+ reduced cell viability to 41.56 ± 1.06% compared to the control group. However, Ukgansan treatment at 50–200 *μ*g/mL prevented differentiated PC12 cell loss induced by MPP+ neurotoxicity, from 57.75 ± 2.81% to 101.05 ± 0.24% (Figure 1(a)). In addition, we checked for programmed apoptosis activity. MPP+ significantly increased the levels of cleaved caspase-3 activity to 210.90 ± 5.19% compared to the control group, whereas treatment with 50–200 *μ*g/kg Ukgansan decreased the MPP+-induced increase in levels of cleaved caspase-3 activity from 206.95 ± 4.56% to 121.76 ± 3.16% (Figure 1(b)).

### 3.2. Effects of Ukgansan on MPP+-Induced Nurr1-Mediated Expression in Differentiated PC12 Cells

**Figure 2:**
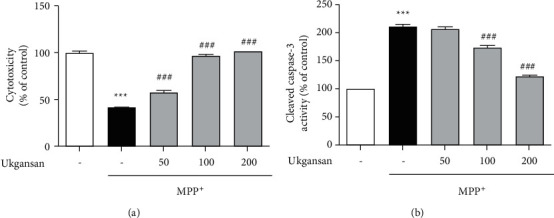
Effect of Ukgansan on MPP+-induced Nurr1 and Nurr1-mediated expression. In differentiated PC12 cells, the effect of Ukgansan on MPP+-induced changed Nurr1-mediated expression (Nurr1, TH, VMAT2, and DAT) levels. Values are presented as means ± S.E.M. ^*∗∗∗*^*p* < 0.001 compared with the control group, ^###^*p* < 0.001 compared with the MPP+-only treatment group.

**Figure 3:**
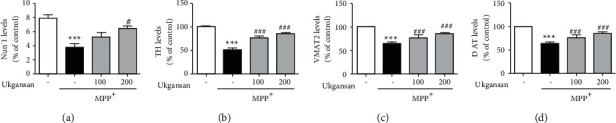
Protective effect of Ukgansan on dopaminergic neurons in differentiated PC12 cells. Dopaminergic neurons were visualized with TH-immunohistochemistry. Representative photomicrographs were taken of SNpc and ST (a) Effect of Ukgansan on changes of neurotransmitter levels in the striatum. ST was dissociated and lysed to measure the dopamine using ELISA kits 7 days after the last MPTP injection (b) Numbers of TH-positive neurons in the SNpc were counted or TH protein levels, and the optical density and TH protein levels in the ST were measured ((c) and (d)). Values are indicated as the mean ± S.E.M. ^*∗∗∗*^*p* < 0.001 compared with the control group, ^##^*p* < 0.01 and ^###^*p* < 0.001 compared with the MPTP-only treated group.

To investigate the effects of Ukgansan on Nurr1 action, we measured the levels of Nurr1-mediated expression in TH, VMAT2, and DAT in differentiated PC12 cells. Treatment with MPP+ reduced the expression levels in Nurr1, TH, VMAT2, and DAT to 7.88 ± 0.44 *μ*M, 51.19 ± 4.39%, 63.53 ± 4.45%, and 71.03 ± 4.92%, respectively, compared to the control group. However, Ukgansan treatment at 100–200 *μ*g/mL increased the levels of expression in Nurr1, TH, VMAT2, and DAT, from 5.17 ± 0.60 to 6.42 ± 0.31 *μ*M, 76.83 ± 3.67% to 85.17 ± 2.35%, from 76.32 ± 6.61% to 85.10 ± 3.38%, from 76.49 ± 2.99% to 88.24 ± 4.59% respectively, in differentiated PC12 cells (Figure 2). Moreover, to further verify whether Ukgansan-induced TH was mediated through Nurr1 activation, we transfected differentiated PC12 cells by targeting siRNA Nurr1. The results confirmed that the TH level in Nurr1 siRNA-infected cells statistically significantly reduced the effect of Ukgansan (Figure 3).

### 3.3. Effect of Ukgansan on MPTP-Induced Movement Impairment

**Figure 4:**
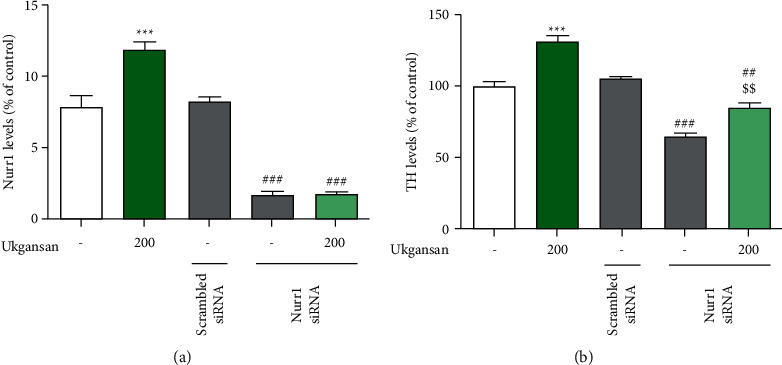
Effect of Ukgansan against MPTP-induced behavioral dysfunction in a mouse PD model. The pole tests were conducted 3 and 7 days after the last MPTP injection. Time to turn downward completely ((a) and (c); T-turn) and arrive at the floor ((b) and (d); T-LA) were recorded with the cut-off limit at 60 s. Values are indicated as the mean ± S.E.M. ^*∗*^*p* < 0.05 and ^*∗*^*p* < 0.01 compared with the control group, ^#^*p* < 0.05, ^##^*p* < 0.01, and ^###^*p* < 0.001 compared with the MPTP-only treated group.

A pole test was performed to evaluate the effect of Ukgansan on the MPTP-induced movement impairment. T-turn and T-LA were significantly prolonged to 187.54 ± 37.65% and 148.51 ± 13.68% on day 3 or to 246.84 ± 40.79% and 144.36 ± 10.20% on day 7, respectively, compared with the control group. However, T-turn and T-LA were significantly shortened in the MPTP + ropinirole and Ukgansan groups to 143.18 ± 15.34% and 84.57 ± 2.44%, 143.89 ± 19.65% and 71.64 ± 6.77% on day 3 or to 109.46 ± 11.20% and 90.68 ± 7.74%, 119.55 ± 18.53% and 117.99 ± 7.82% on day 7, respectively, as compared with controls (Figure 4).

### 3.4. Effects of Ukgansan on MPTP-Induced Dopaminergic Neuronal Loss and Dopamine Depletion

To confirm the effect of Ukgansan on dopaminergic neuronal loss and dopamine content, we performed TH immunohistochemistry in the SNpc and ST and striatal dopamine levels in mouse brains. In the MPTP-only treated mice, the number of TH-positive cells in the ST and optical intensity in the SNpc decreased to 43.98 ± 10.54% and 53.49 ± 7.54%, respectively, compared with the control group. However, Ukgansan treatment significantly increased these treatments to 80.39 ± 2.98% and 88.82 ± 5.63%, respectively (Figures 3(a)–3(c)). Moreover, treatment with MPTP significantly decreased striatal dopamine to 2.23 ± 0.18 *μ*M/L compared with the control group, while treatment with 200 mg/kg Ukgansan reduced MPTP-induced striatal dopamine to 4.808 ± 0.35 *μ*M/L (Figure 3(d)).

### 3.5. Effects of Ukgansan on MPTP-Induced Nurr1 Expression and Its Nurr1-Mediated Expression

**Figure 5:**
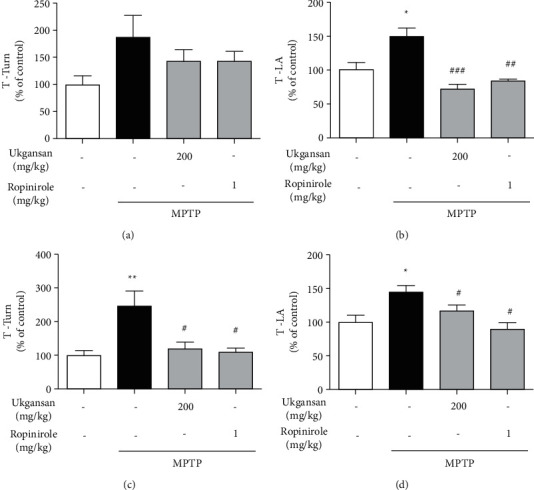
Effect of Ukgansan on MPTP-induced Nurr1 expression. The effect of Ukgansan on changes of Nurr1 expression levels in the SNpc 7 days after the last MPTP treatment. Values are presented as means ± S.E.M. ^*∗∗∗*^*p* < 0.001 compared with the control group, ^###^*p* < 0.001 compared with the MPTP-only treatment group.

**Figure 6:**
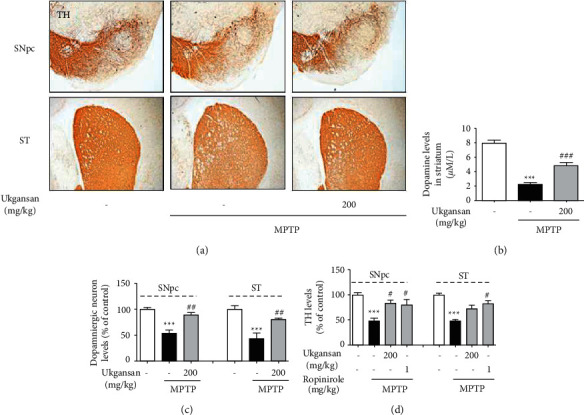
Effect of Ukgansan on MPTP-induced Nurr1-mediated expression. The effect of Ukgansan on changes of Nurr1-mediated expression (TH, VMAT2, and DAT) levels in the striatum 7 days after the last MPTP treatment. Values are presented as means ± S.E.M. ^*∗*^*p* < 0.05 and ^*∗*^*p* < 0.01 compared with the control group, ^#^*p* < 0.05 and ^##^*p* < 0.01 compared with the MPTP-only treatment group.

To evaluate the effects of Ukgansan on Nurr1 expression, Nurr1 immunoreactivity in the SNpc of the brain was examined using western blotting. Treatment with MPTP significantly decreased Nurr1 to 29.53 ± 2.80% compared with the control group, while treatment with 200 mg/kg Ukgansan reduced MPTP-induced Nurr1 to 59.01 ± 4.18% (Figure 5). To measure the effects of Ukgansan on MPTP-induced Nurr1-mediated expression, we assessed TH, VMAT2, and DAT levels in the ST of the brain. Treatment with MPTP significantly decreased TH, VMAT2, and DAT to 57.79 ± 6.81%, 31.78 ± 9.45%, and 47.93 ± 17.01% compared with the control group, while treatment with 200 mg/kg Ukgansan reduced MPTP-induced TH, VMAT2, and DAT to 94.66 ± 3.36%, 78.93 ± 10.03%, and 106.57 ± 4.65% (Figure 6).

## 4. Discussion

**Figure 7:**
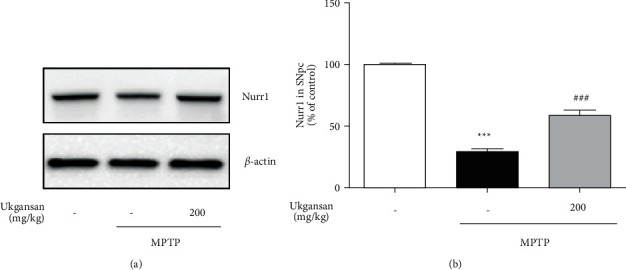
Summary of the experimental design. Differentiated PC12 cells and mice were treated with Ukgansan or ropinirole before MPP+ and MPTP induced the toxicity. Pole test was performed on day 3 and 7 after the last injections of MPTP. Differentiated PC12 cells were analyzed 23 h after stimulation of MPP+, and the brain tissues of mice were analyzed 7 days after the last injections of MPTP.

MPTP, a neurotoxin used in PD mouse models, can induce PD symptoms similar to those in human PD [22]. Therefore, it is useful for therapeutic research by revealing the molecular and cellular mechanisms underlying PD [22, 23]. MPTP is lipophilic and easily passes through the blood-brain barrier (BBB). It is then metabolized to the toxic metabolite 1-methyl-4-phenylpyridinium (MPP+) ion by monoamine oxidase type B (MAO-B) in astrocytes, which triggers cell damage to the ST and SNpc [23, 24]. In addition, MPTP causes cell death of dopaminergic neurons through mitochondrial apoptosis, oxidative stress, alpha-synuclein production, excessive binding of glutamate, and inflammation [24]. Ukgansan was able to predict the neuronal protective effects based on previously reported results [11]. However, this was verified using differentiated PC12 cells to identify changes in dopaminergic neurons directly [25–27]. To confirm the neuroprotective effect of Ukgansan against MPP+-induced toxicity, cytotoxicity, cleaved caspase-3 activity, and Nurr1-mediated expression were investigated in differentiated PC12 cells. As shown in Figure 7, Ukgansan inhibited programmed apoptosis signaling by blocking PC12 cell loss and the activity of cleaved caspase-3 compared to the MPP+-only treated group in differentiated PC12 cells.

**Figure 8:**
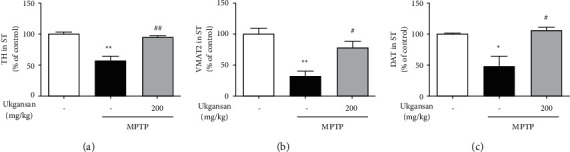
Effects of Nurr1 on upregulating Nurr1-mediated expression (TH) in Nurr1 siRNA-transfected differentiated PC12 cells. Values are presented as means ± S.E.M. ^*∗∗∗*^*p* < 0.001 compared with the control group, ^##^*p* < 0.01 and ^###^*p* < 0.001 compared with the scrambled siRNA-only treatment group, and ^$$^*p* < 0.01 compared with the siNurr1-only treatment group.

MPTP also induces movement disorders by selectively destroying dopaminergic neurons in the substantia nigra and decreasing dopamine terminals from the ST [28]. To confirm the effects of Ukgansan on motor symptoms, dopaminergic neurons, and dopamine content in PD, the pole test, dopaminergic neuronal loss in the SNpc and ST, and dopamine levels in the ST were investigated in a mouse model. Since ropinirole, a dopamine agonist, has been shown to protect dopaminergic neurons against MPTP-induced neurotoxicity and improve movement impairment, it was used as a positive control for Ukgansan [29–32]. In mouse PD models, the pole test has been used in various studies to evaluate movement disorders, such as bradykinesia [33]. As shown in Figure 2, both ropinirole-treated and Ukgansan-treated mice had shortened T-turn and T-LA compared to MPTP-only treated mice, which improved movement impairment. The results of the behavioral experiments were also similar to those of previous studies [11, 12]. In addition, as shown in Figure 8, Ukgansan prevented MPTP-induced loss of dopaminergic neurons in the SNpc and ST and reduced dopamine depletion in ST, suggesting that Ukgansan protects dopaminergic neurons and maintains dopamine released to the ST, thereby improving movement disorders. Changes in the amount of dopamine in PD are very important, but no research results have been reported; however, this study obtained the results.

Nurr1 is decreased in dopaminergic neurons of the substantia nigra (SN) with pathological signs of PD in brain tissue [6, 34]. It is also correlated with TH loss, an enzyme that plays an important role in dopamine synthesis and catalyzes the hydroxylation of tyrosine to L-DOPA [6]. Nurr1 expression was decreased in the peripheral blood lymphocytes of patients with PD. Decreased Nurr1 function increases PD progression and severity [6]. In addition, disruption of Nurr1 expression also decreased the TH, VMAT2, and DAT genes associated with dopaminergic neurons [35, 36]. VMAT2, which transports dopamine from the cytoplasm to the synaptic vesicles, and DAT, which reuptakes dopamine released from the synaptic cleft, play important roles in dopamine homeostasis [35, 36]. In this study, as shown in Figure 1, Ukgansan prevented the MPP+-induced decrease in levels of Nurr1 and its-related TH, VMAT2, and DAT in differentiated PC12 cells. In addition, it was confirmed that the TH level in Nurr1 siRNA-infected cells statistically significantly reduced the effect of Ukgansan. However, experimental results show that the Ukgansan does not regulate dopamine cells by utilizing only the activity of Nurr1, but also by activating other mechanisms, and related reports have been reported on various mechanisms [11–16]. For example, Ukgansan protected dopaminergic neurons through antioxidant effects via the activity of Nrf2. In addition, there is a report that Ukgansan increased the efficiency of dopamine activity through the effect of improving dopamine receptors' agonistic action [11, 12]. To confirm the effects of Ukgansan on Nurr1 and its regulating factors, Nurr1 expression in the SNpc and the levels of TH, VMAT2, and DAT in the ST of mice brains were investigated. As shown in Figures 3 and 5, Ukgansan increased Nurr1 expression in the SNpc and the levels of TH, VMAT2, and DAT in the ST compared to the MPTP-only treatment group. Nurr1 promotes the survival of dopaminergic neurons in MPTP-induced toxicity [35, 36]. Likewise, Nurr1 overexpression had a neuroprotective effect on cell death induced by MPTP-induced neurotoxicity [36]. The expression of Nurr1 is correlated with nigral dopaminergic neuronal function, associated with changes in locomotor activity due to a decrease in dopamine levels [37]. Specifically, reductions in locomotor performance correlated with striatal dopamine and Nurr1 mRNA levels. Nurr1-like agonists that mimic the transcriptional activity of Nurr1 have been found to enhance TH and DAT in animal models of PD [38]. Similarly, pramipexole, a dopamine receptor agonist, enhanced the expression of Nurr1 signaling [39].

Various studies have been conducted on the effects and mechanisms of Ukgansan and its herbal ingredients to confirm its potential application in the treatment of neurological diseases, including PD [40]. It was also reported that the multiple potential actions of Ukgansan in PD were explained through neurotransmissions, such as glutamate and dopamine, and mechanisms such as neuroprotection, neuroplasticity promotion, and the anti-effects of stress and inflammation [17, 21, 40]. Ukgansan was reported to have neuroprotective effects on dopaminergic neurons from toxicity caused by MPP+/MPTP and improved motor function by activating phosphatidylinositol 3-kinase (PI3K)/Akt. Ukgansan increased the therapeutic effect of L-DOPA by inhibiting catechol-O-methyltransferase (COMT) in a PD model induced by 6-hydroxydopamine (6-OHDA) [12, 21]. Ukgansan protected dopaminergic neurons and improved movement disorders in the same PD model by assisting with L-DOPA [12]. Ukgansan also inhibits dyskinesia, a side effect of L-DOPA, by reducing the overexpression of proteins related to dopamine receptor 1 (D1R) [12]. In addition, Ukgansan prevented the loss of dopaminergic neurons and alleviated motor dysfunction by activating nuclear factor erythroid 2-related factor 2 (Nrf2), which promotes antioxidant action in a 6-OHDA-induced PD model [11]. However, no studies have reported that Ukgansan can activate Nurr1, which plays an essential role in the survival of dopamine neurons and the production and maintenance of dopamine. Maintaining dopamine in PD and subsequent behavioral changes is very important; thus, this study confirms new research results.

Ukgansan was characterized as a total of 64 compounds: twenty-three triterpene saponins, sixteen flavonoids, eleven phenolic compounds, eight alkaloids, and six other compounds. Analysis of the proportion of each herb for 64 compounds extracted from Ukgansan revealed that Glycyrrhizae Radix et Rhizoma, Uncariae Ramulus cum Uncus, and Cnidii Rhizoma were the most important ingredients. Furthermore, the amount of seven major compounds with antioxidant, anti-inflammatory, and neuroprotective effects, which have already been revealed through research on herbs constituting Ukgansan, increased when the decoction was extracted by combining the herbs of Ukgansan, compared to single-plant decoction [41]. Therefore, to investigate the effect and mechanism of Ukgansan used for PD treatment in the clinical field, it would be effective to use Ukgansan in the form of a mixed decoction, as in this study.

However, since this study was conducted in vitro and in vivo, there is a limitation in that it is only possible to estimate whether Ukgansan is effective in patients with PD. Nevertheless, in a study on the side effects of Ukgansan for up to 52 weeks in 3156 patients in Japan, 136 cases (4.3%) of side effects were reported, confirming that the use of Ukgansan was relatively safe and can be used in clinical studies [42]. Therefore, based on previous studies on Ukgansan, a prospective study with a high level of evidence on the relationship between Ukgansan and Nurr1 in patients with PD is expected to be conducted in the future.

Altogether, these results suggest that Ukgansan regulates the Nurr1 signaling pathway in a mouse PD model to protect dopaminergic neurons from MPTP-induced neurotoxicity and increase Nurr1 regulatory factors, thereby preventing dopamine depletion and improving movement impairment.

## Figures and Tables

**Table 1:** Components of ukgansan.

Latin name	Amount (g)
Atractylodis rhizoma	40
Poria sclerotium	40
Cnidii rhizoma	30
Angelicae gigantis radix	30
Uncariae ramulus cum uncus	30
Bupleuri radix	20
Glycyrrhizae radix et rhizoma	15
Total amount	205

## Data Availability

The data used to support the findings of this study can be obtained from the corresponding author upon request.
